# Do Aphids Alter Leaf Surface Temperature Patterns During Early Infestation?

**DOI:** 10.3390/insects9010034

**Published:** 2018-03-14

**Authors:** Thomas Cahon, Robin Caillon, Sylvain Pincebourde

**Affiliations:** Institut de Recherche sur la Biologie de l’Insecte, UMR 7261, CNRS, Université de Tours, 37200 Tours, France; thomas.cahon@etu.univ-tours.fr (T.C.); robin.caillon@etu.univ-tours.fr (R.C.)

**Keywords:** *Aphis pomi*, apple, herbivory, leaf temperature, stomatal behavior, thermal heterogeneity, thermography

## Abstract

Arthropods at the surface of plants live in particular microclimatic conditions that can differ from atmospheric conditions. The temperature of plant leaves can deviate from air temperature, and leaf temperature influences the eco-physiology of small insects. The activity of insects feeding on leaf tissues, may, however, induce changes in leaf surface temperatures, but this effect was only rarely demonstrated. Using thermography analysis of leaf surfaces under controlled environmental conditions, we quantified the impact of presence of apple green aphids on the temperature distribution of apple leaves during early infestation. Aphids induced a slight change in leaf surface temperature patterns after only three days of infestation, mostly due to the effect of aphids on the maximal temperature that can be found at the leaf surface. Aphids may induce stomatal closure, leading to a lower transpiration rate. This effect was local since aphids modified the configuration of the temperature distribution over leaf surfaces. Aphids were positioned at temperatures near the maximal leaf surface temperatures, thus potentially experiencing the thermal changes. The feedback effect of feeding activity by insects on their host plant can be important and should be quantified to better predict the response of phytophagous insects to environmental changes.

## 1. Introduction

Habitat temperature is one of the most influential abiotic factors driving the distribution and abundance of organisms, because it influences virtually all biochemical and physiological rates [1,2]. Thus, the direct effect of temperature on the distribution of organisms at both the geographical and local scales has received considerable attention. The classical approach consists in linking biological patterns and average habitat temperature, usually being averaged both in time (e.g., monthly average) and space (e.g., across the microhabitat). Nevertheless, not only does the mean of temperature matter, but also its variance across space [3,4,5,6] and time [7,8,9] is also important. The spatial variance in temperature is especially important for thermoregulating ectotherms since it determines the thermal opportunities in terms of favorable and risky patches [10]. The link between spatial variance (or microclimate heterogeneity) and its impacts on ectotherms has been traditionally studied at scales above meters [11]. Strikingly, little is known about the impact of atmospheric temperature on the spatial heterogeneity of temperatures at very fine scales that are relevant for small arthropods (see [12] for a notable exception), such as individual leaf surfaces, despite the great diversity of organisms living in the leaf microhabitat [13].

Single leaf surfaces can show substantial thermal heterogeneity [14,15,16,17]. This spatial heterogeneity is generated through three biophysical processes underlying heat exchanges between the plant and the environment [13,16,18]: (i) the boundary layer, which is the air layer at the interface between the leaf and nearby free-stream air; (ii) the stomatal patchiness, which is the spatial and temporal heterogeneity of the stomatal conductance over a single leaf surface [14,19]; and (iii) the interaction between irradiance and leaf microtopography, which generate variability in surface temperature according to the inclination of each leaf portion relative to the sun position [16]. The thickness of the boundary layer can reach 10 mm depending on wind speed, the size, and shape of the leaf and the density of trichomes at its surface [18,20]. Stomatal patchiness modifies local conditions within the boundary layer of a leaf [21]. Small arthropods, such as aphids or insect eggs (<1 mm), are directly influenced by the temperature deviations between leaf surface temperature and air temperature [22]. Small arthropods can exploit the thermal heterogeneity of the leaf microclimate to perform behavioral thermoregulation within a single leaf surface [23,24], although these studies quantified the leaf surface temperature heterogeneity when the insect was not feeding on the plant.

The feeding activity of insect herbivores generates various types of damage to plant tissues. This damage can greatly affect leaf gas exchange and water status [25,26,27]. In general, insect herbivores reduce the photosynthetic rate when feeding on leaf tissues [28,29,30], while the transpiration rate can be increased or decreased depending on the herbivore species [31,32]. These effects likely have consequences for the leaf surface temperature heterogeneity. For example, thermographic studies visualized the very local (within the leaf surface) effect of insect feeding on the leaf surface temperature [32,33]. Notably, an increase in surface temperature was observed in general around the exact location of the insect, but these studies did not report a quantitative assessment of the surface temperature pattern at the whole leaf scale. Therefore, the quantitative consequences of such effects on the leaf surface temperature heterogeneity remain unclear. In particular, the extent to which herbivores may induce an increase in the mean leaf temperature and on metrics of thermal heterogeneity was not determined.

Here, we measured the impact of aphids on the leaf surface temperature heterogeneity with a thermographic approach. Aphids are small enough to remain embedded within the leaf boundary layer (see [23]) and to depend upon variations in leaf surface temperature [13]. Using thermal images of upper leaf surfaces that were taken at different air temperatures, we compared the leaf surface thermal heterogeneity of intact leaves and leaves infested with the green apple aphid, *Aphis pomi*. In addition, leaves that were infested by aphids were compared to leaves with the underside fully covered with vegetable oil to inhibit transpiration via the stomata. This comparison allowed us to infer the mechanisms at play when aphids induce shifts in the leaf surface temperature distributions. To our knowledge, the effect of this aphid species on the transpiration rate of its host plant was never been determined; other aphid species were found to increase or decrease the transpiration rate [34,35,36]. We hypothesized that the impact of aphids is local and should lead to a change in the configuration of the leaf surface temperature distribution. We recorded the position of aphids at the lower leaf surface relative to the upper surface temperature pattern to explore the question of how the aphid uses its thermal environment. Although these aphids are found on the lower leaf side, measuring thermal profiles from the upper leaf surface is easier than from the lower leaf side and the surface temperature of the two apple leaf sides do not differ [24]. 

## 2. Materials and Methods

### 2.1. Plants and Insects

Leaf surface temperature measurements were made on the leaves of *Malus domestica* cv Golden, (Rosaceae). Apple seedlings (<3 years old) were grown in pots (15 cm in diameter, volume 1.2 L) in a greenhouse with variable meteorological conditions. Air temperature ranged from 14 °C to 42 °C and air relative humidity from 29 to 95% during the study period in spring 2014. Seedlings were watered generously about every 2–3 days, according to conditions, and each pot received nitrogen-enriched fertilizer (N-P-K: 5-3-7). The plants never suffered water stress. The plants were distributed between two groups that were isolated from each other in the greenhouse facility: the first corresponded to intact plants free of any pest, and in the second group the plants were infested with aphids. The second group provided aphids to infest the plants from the first group at the time that was needed for the experiments (see below). Apple seedlings were infested with the green apple aphid, *Aphis pomi* (Hemiptera: Aphididae). This herbivore species is a specialist on the domesticated apple. The colonies originated from females collected in the field near the laboratory a year before (2013). Only adult females were used in the experiments. 

### 2.2. Experimental Design

We quantified the effect of presence and feeding activity of aphids on the leaf surface temperature distribution in interaction with ambient air temperature. The experimental design consisted in placing apple seedlings within a climatic chamber (VB 1014-A, Vötsch, Balingen, Frommern, Germany) set with a gradual change in air temperature during six hours, by recording the surface temperature of a leaf every 30 min using an infrared camera that was positioned above the plant. Due to practical constraints, the plants were experimented one by one (i.e., one plant per day) within the climatic chamber. Apple seedlings were placed within the climatic chamber 18 h before the experiment to homogenize their physiological state. The conditions during this acclimation procedure were: photoperiod 11:13 (L:D), relative humidity 60% and air temperature 15 °C. Then, the six-hour experimental procedure started at 10:00 with a linear increase in air temperature from 15 °C to 30 °C during 3 h, followed by a linear decrease back to 15 °C at the end of the experiment at 16:00. This air temperature pattern was the best compromise between the technical constraints of the climatic chamber and the need to reproduce a variation that is close to what could be observed in the field.

**Figure A1:**
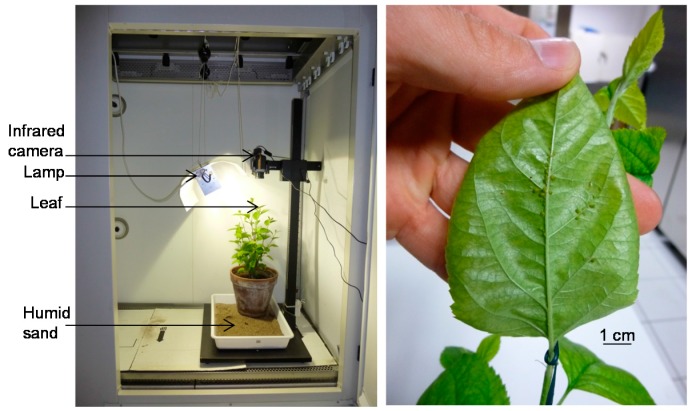
(**Left**) The experimental setup. The plant (apple seedling) was positioned within a climatic chamber, below a lamp slightly shifted aside such that the infrared camera could be placed directly above the leaf surface (exactly perpendicular). The level if irradiance was measured by placing the radiation sensor exactly at the location of the focal leaf. The humid sand was used to create a contrasting background on the IR images. (**Right**) A photograph of the leaf surfaces was taken before and after the six-hour experimental period to determine the location of aphids at the leaf surface and to verify that they did not move during the experiment. Photographs: Thomas Cahon.

During each six-hour experimental period, a different apple seedling was used and a single leaf was followed with an infrared camera (FLIR Systems, B335, Wilsonville, OR, USA), equipped with a macro lens (FLIR Systems, 10 mm diameter). Apple leaves were selected at the age of about 30 days as their photosynthetic and transpiration rates were found to be maximal at this age [37]. Emissivity of the camera was fixed to 0.99 [24]. The thermal camera was positioned directly above the leaf surface at a distance of about 30 cm. This fixed distance allowed for us to avoid the distance effect on the camera readings [38]. Previous studies showed that the upper and lower surfaces of an apple leaf have similar surface temperature patterns [16,24]. In the climatic chamber, the plant was positioned such that the focal leaf was almost flat relative to the lamp (hydrargyrum medium-arc iodide lamp, HSI-T SX 400 W, Sylvania Britelux, Cityplants, Paris, France). The plant was placed at a distance of 20–25 cm from the lamp so the focal leaf received 230–250 W·m^−2^ of irradiance (the difference of 20 W·m^−2^ in one replicate should not cause a leaf surface temperature deviation of more than 0.2 °C; see the model tested in the same conditions in [39]). The lamp and the camera were necessarily close to each other (Figure A1). The lamp cannot be considered as a point source, but as a distributed source: lamp extension was therefore characterized by specifying (from pictures of the setup) two extreme elevation angles from which light beams come from and hit the leaf surface, here 18° and 64° (see [16] for more information on the same setup). Wind regime was turbulent within the climatic chamber, i.e., coming from all directions at the scale of the leaf and with a speed of 0.4 m·s^−1^. This regime implies that colder surface temperatures are expected to be located at the periphery of the leaf and all around the leaf. A thermographic image of the entire leaf surface was taken every 30 min. 

Leaf surface temperatures were measured on three groups of plants. A total of 15 plants were used, and only one leaf per plant was measured (*n* = 15 leaves total). The first group (*n* = 5 plants) corresponded to leaves infested with aphids. One leaf per plant was infested with 15 females three days before the experiment to ensure a reasonable population size during infrared measurements (between 30 and 50 individuals), while avoiding any influence of the aphids on the leaf shape (e.g., curling, rolling). In the second group (*n* = 5 plants), the lower side of one apple leaf, which contains stomata, was fully covered with vegetable oil following the method in [27]. This treatment inhibited the transpiration rate [27] and allowed for an estimation of the leaf surface temperature distribution when the transpiration rate is near zero. The third group (*n* = 5 plants) was the control: intact leaves without any treatment. Globally, the same sequence of treatments was repeated throughout the experimental period, with the intact leaf treatment on day 1, followed by the vegetable oil (day 2) and the aphid (day 3) treatments. The conditions in the greenhouse were similar enough between days to ensure that individual plants were comparable.

The number of aphids and their position at the leaf surface was measured. The green aphid usually lives on the lower apple leaf surface close to the main and secondary veins. A photograph of the lower leaf surface was taken just before and again at the end of the six-hour experiment. The picture at the end of the experiment was analyzed to describe the position of every individual that was detected in a two-dimensional (2D) coordinate system with the basis of the petiole as origin and the *X*-axis along the beginning of the main vein (in ImageJ 1.47v, Wayne Rasband, NIH, USA). The same procedure was applied to the IR image to retrieve the leaf surface temperature at the exact position of all the aphids that were detected in the photograph (at least 30 individuals were detected on the photographs—too young or aggregated individuals were impossible to detect from the photographs). This procedure assumes that the individuals did not move across the leaf surface during the experiment as the air temperature varied. Indeed, we did not observe any difference in the position of the aphids between the two pictures taken at the beginning and the end of the experiment—except for the presence of individuals born during the experiment.

### 2.3. Statistical Analysis

For each leaf and each time step (every 30 min during 6 h), the temperature of each pixel of the leaf surface in the thermographic image was extracted using ThermaCam Researcher Professional (FLIR Systems). This procedure was used to collect the temperature distribution for each single leaf at each time step. Then, various variables were computed to study the thermal heterogeneity of leaf surfaces in terms of composition and configuration. The composition was studied by calculating the minimal (defined as the mean temperature of the coolest 5% of pixels at the surface), maximal (defined as the mean temperature of the hottest 5% of pixels of the surface), and mean temperature (considering all the pixels of the leaf surface, the number of which ranged between 3130 and 7744 pixels in our sample of 15 leaves) from the distributions for each leaf at each time step. Results were unchanged qualitatively when taking the single coolest and hottest pixels for minimal and maximal surface temperature, respectively (see Results). In addition, from these three metrics, we calculated the surface temperature excess as the difference between the surface and ambient air (i.e., leaf surface temperature minus air temperature) in order to standardize for air temperature and to infer the behavior of the leaf surface temperature patterns according to air temperature. 

Then, the patch richness density (PRD) was computed as a complement to the simple temperature range. The PRD corresponds to the number of patch types standardized to a per area basis: in our case, the PRD is the count of different temperature values (with a 0.1 °C resolution) in the thermographic image, divided by the total leaf area. The PRD is given as the number of patches (or temperature values) per unit of leaf surface (in cm^2^). It is close to zero when the diversity of temperature values is small, and it increases when the temperature distribution is broadened (i.e., when every single pixel has a unique temperature value). For the configuration of the thermal heterogeneity, we calculated the aggregation index (AI), which defines the way groups of pixels with similar temperatures are arranged spatially [24]. The AI is computed by dividing the number of adjacencies between pixels of same temperature and the maximum number of adjacencies between those pixels obtained if all the pixels of similar temperature were grouped together (see formulae given in Fragstat: [40]). The AI (multiplied by 100 to convert values to percentage) is zero when similar pixels are spread across the surface, and it equals 100 when the aggregation is maximal—the surface therefore appears patchy with all of the similar pixels grouped together [41]. Both the PRD and the AI were computed using Fragstats (v4.2, 2013, [40]).

The effects of air temperature and treatment (vegetable oil versus aphids versus control) on leaf surface temperature patterns were tested with an analysis of covariance (ANCOVA), with the treatment as a fixed factor, air temperature as a covariate and leaf identity as a random factor that is nested in the treatment. The residuals of the models were checked visually to make sure of their linearity, and the homoskedasticity of the data was verified with the Breuch Pagan test. Interaction terms included the interaction between air temperature and treatments. Finally, we analyzed the position of aphids relative to the leaf surface temperature distribution using two-sample t-tests for comparing the two distributions for each replicate. The different metrics of aphids (their deviation to maximal or mean leaf temperature) were compared to a theoretical distribution that was centered on 0 and with a confidence interval of 0.9 using one-sample t-tests with a Dunn-Sidak correction for multiple comparisons. All of the analyses were done using R software (v3.1.0, [42]). 

## 3. Results

### 3.1. Leaf Temperature Heterogeneity: Composition

**Figure 1:**
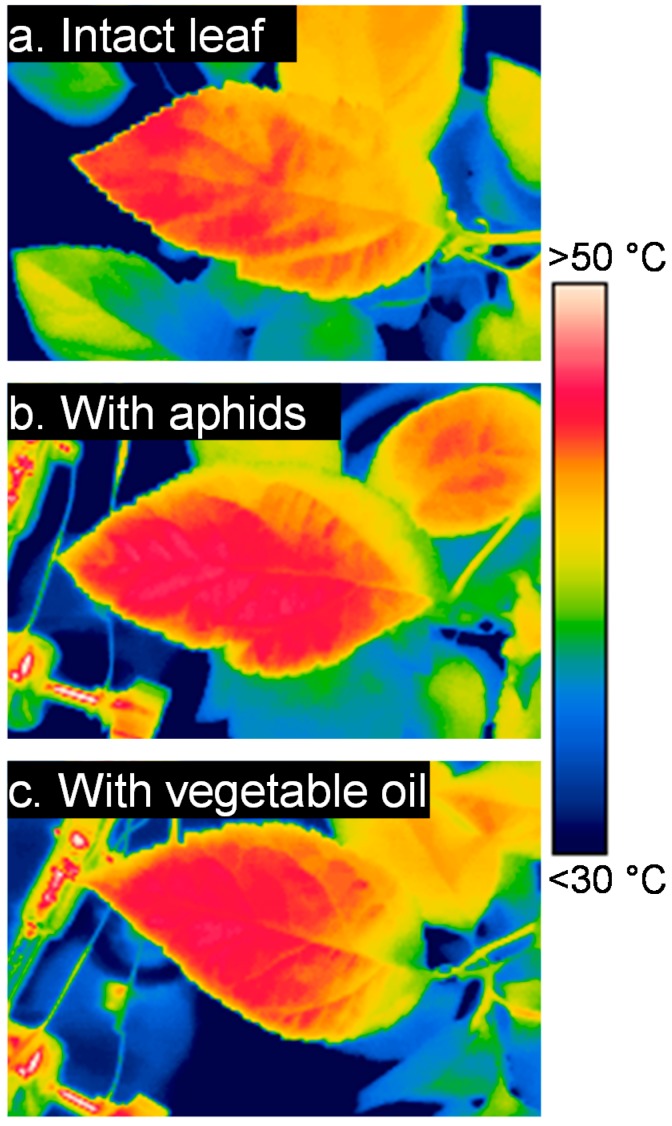
Example of three infrared images showing an intact apple leaf surface (**a**), a leaf with aphids on the adaxial (i.e., lower) apple leaf surface (**b**) and a leaf with the adaxial surface covered with vegetable oil to inhibit evapotranspiration (**c**). These images were taken at air temperature 29 °C and irradiance level 230 W·m^−2^. The same color scale was set to the three images.

**Figure 2:**
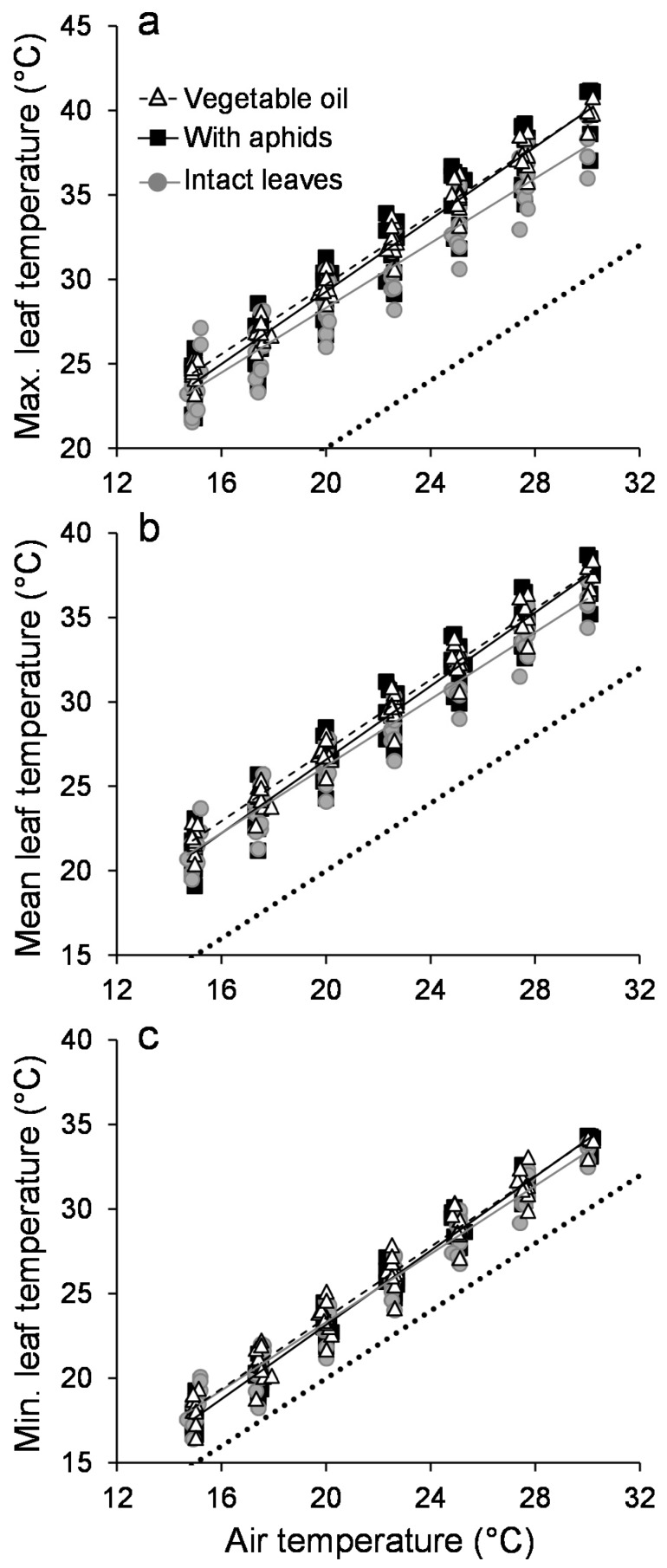
Composition of the leaf surface thermal heterogeneity. Dynamics of (**a**) the maximal leaf temperature, (**b**) the mean leaf temperature and (**c**) the minimal leaf temperature as function of air temperature for intact leaves (grey circles), leaves covered with vegetable oil (white triangles) and leaves infested with green apple aphids (black squares). Lines represent linear regression models for illustrating the trends. The dotted line represents the equality line. The minimal and maximal temperatures correspond to the mean temperature of the 5% coolest and hottest pixels at the leaf surface, respectively.

**Figure A2:**
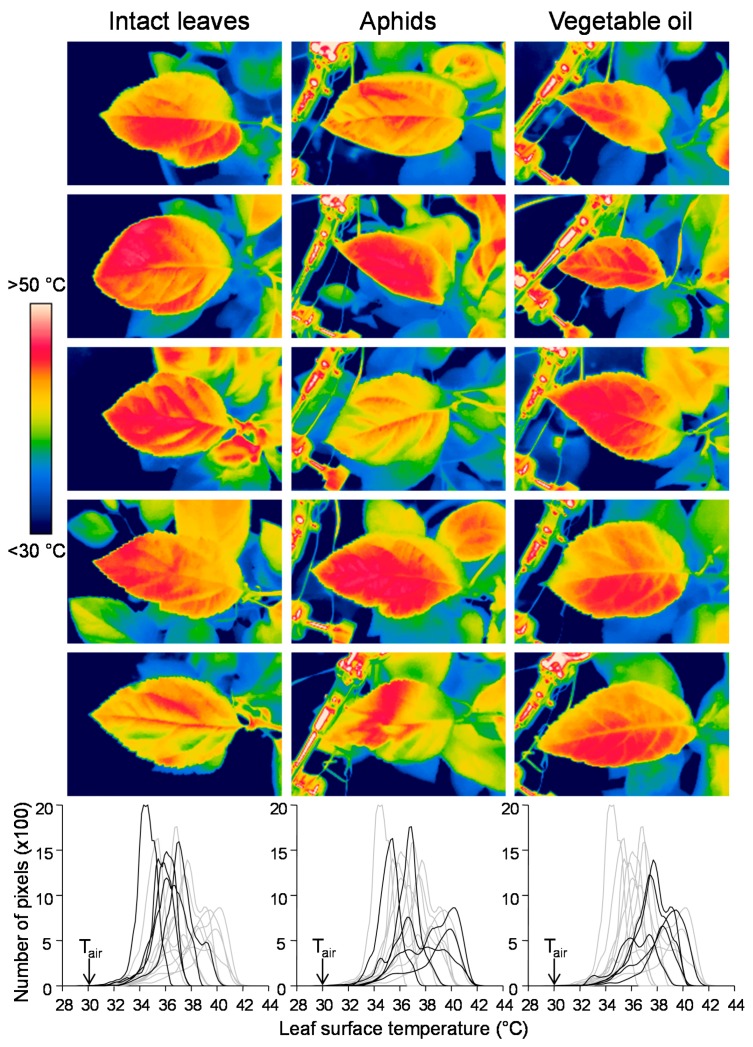
Illustration of the raw data we obtained with our experimental design. The infrared images for each replicate (5 per treatment) and each treatment (intact leaves, leaves with aphids and leaves covered with vegetable oil) were taken every 30 min during 6 h—here the infrared images were taken at 13:30 when air temperature was 30 °C. The color scale is the same for all images. The distributions at the bottom of each column of infrared images show the leaf surface temperature distributions for each replicate in the treatment (corresponding black lines). The grey lines are the distributions for the replicates of the two other treatments, for a visual comparison. These distributions are shown as kernels to better visualize the asymmetry of the curves and sometimes their bimodality. These distributions are shown according to the number of pixels in each temperature bin with a resolution of 0.5 °C. Arrows on the *X*-axis indicate air temperature. Note the strange shape of the second leaf (from the top) for the aphid treatment: the leaf was asymmetric with left limb narrower than the right limb, and the edge started to curl.

**Figure A3:**
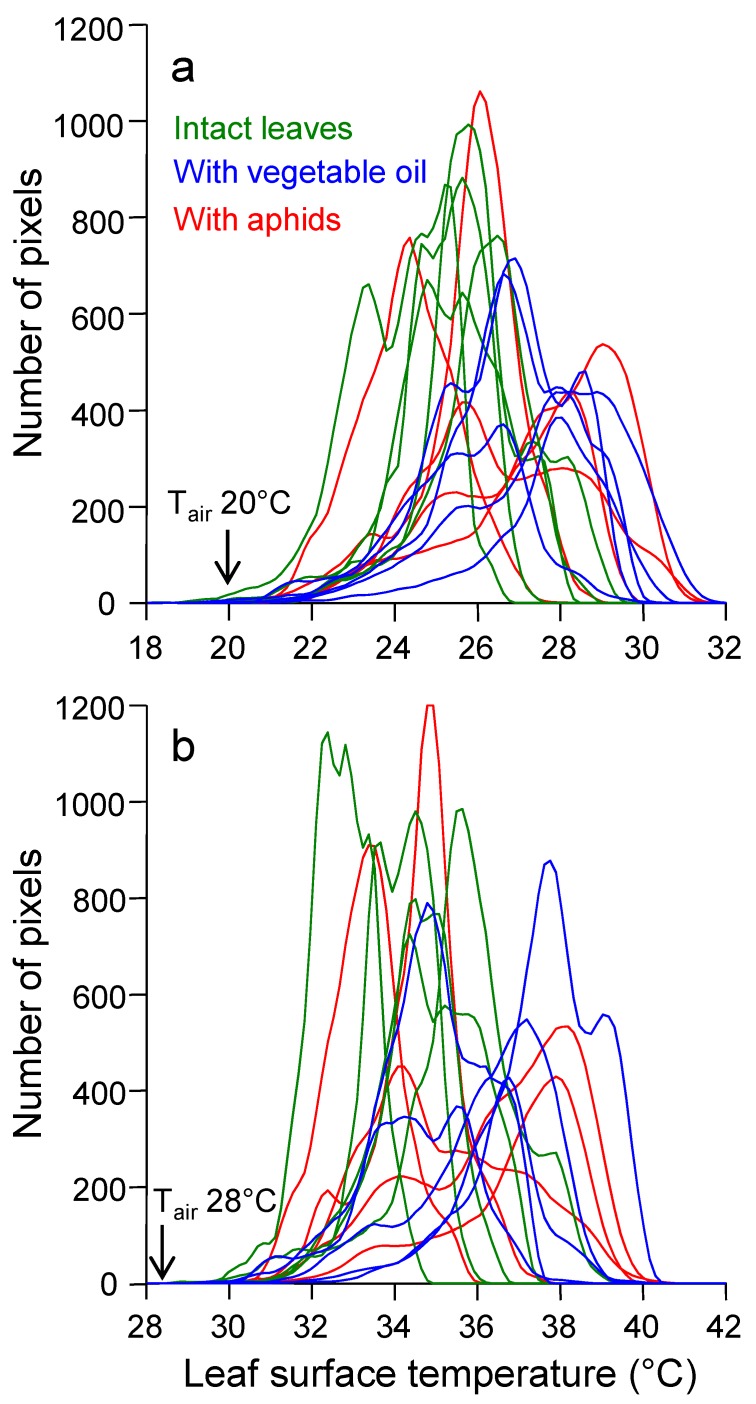
Leaf surface temperature distribution of each leaf in the three treatments at air temperature (**a**) 20 °C (at 11:00) and (**b**) 28 °C (at 13:30): intact leaves (green), leaves covered with vegetable oil to inhibit transpiration (blue) and leaves infested with green apple aphids (red). Each curve is the kernel distribution for a single leaf and it shows the number of pixels per temperature bin (every 0.1 °C). Kernel distributions allowed us to visualize the asymmetry and sometimes the bimodality of the distributions. The arrows on the *X*-axis indicate the air temperature values.

**Figure A4:**
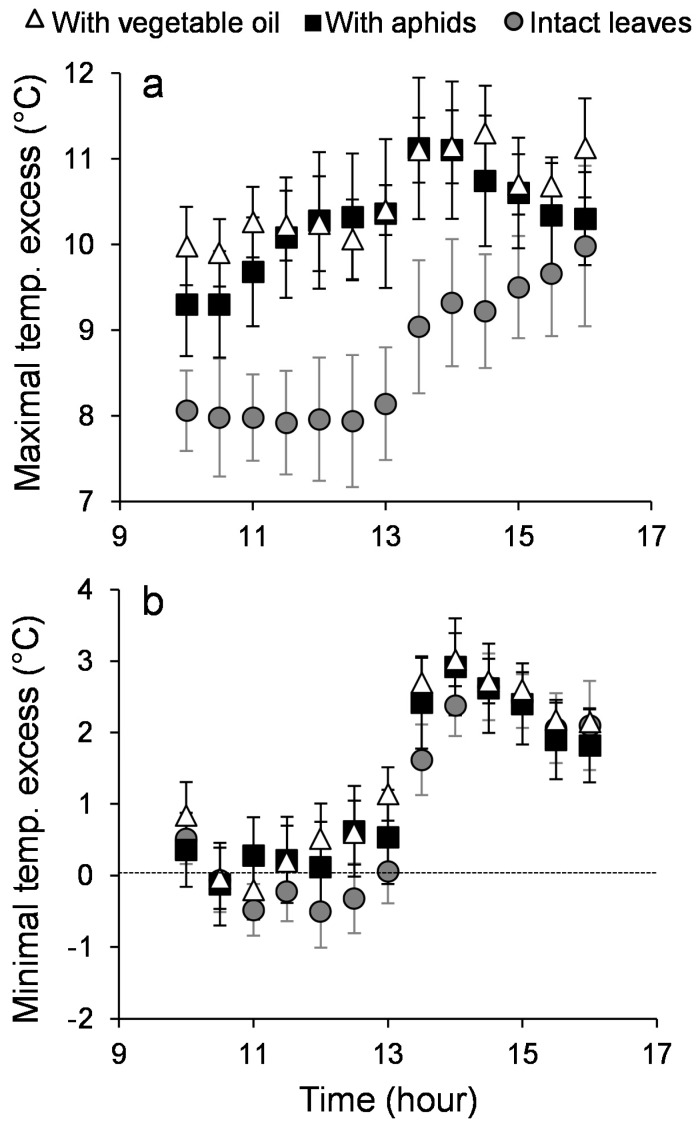
Dynamics of the temperature patterns during the six-hour experiment. Dynamics of the leaf surface temperature excess for the maximal (**a**) and minimal (**b**) leaf temperatures (mean+se for each time step, i.e., every 30 min), and for the three treatments: intact leaves (grey circles), leaves covered with vegetable oil (white triangles) and leaves infested with green apple aphids (black squares). The maximal and minimal temperatures were taken as the hottest and coldest pixels at the leaf surface, respectively.

**Figure A5:**
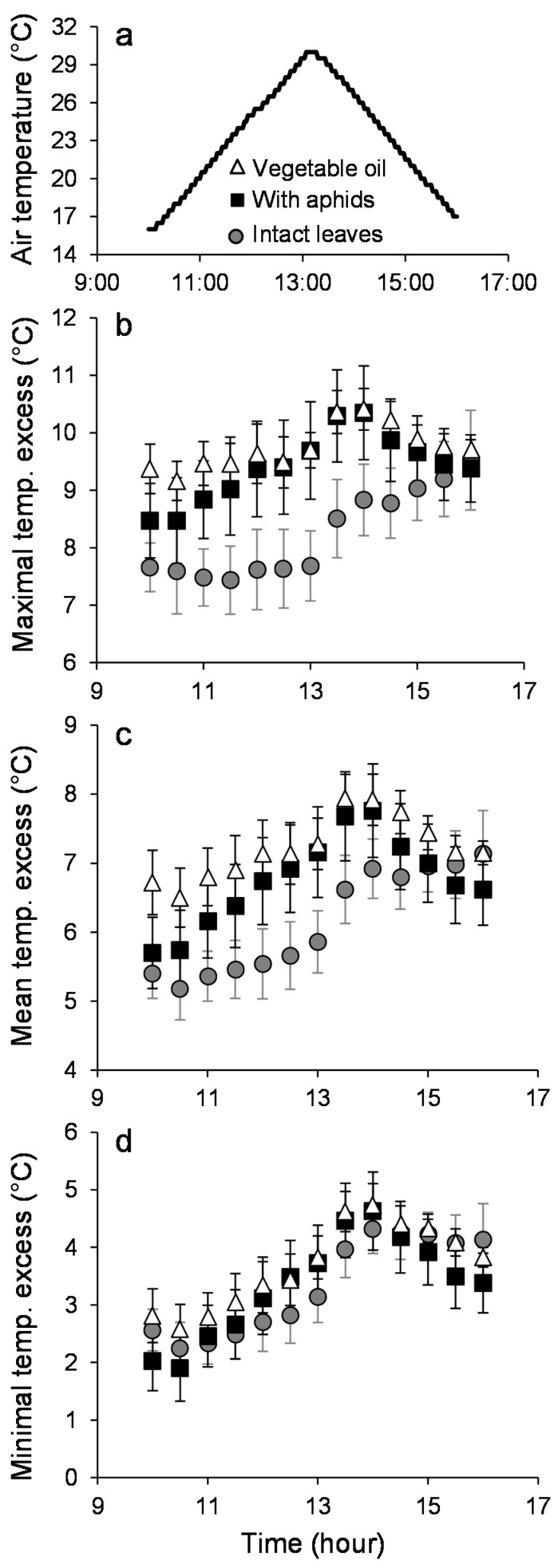
Dynamics of the temperature patterns during the six-hour experiment. (**a**) Air temperature pattern in the climatic chamber from 11:00 to 16:00. (**b**–**d**) Dynamics of the leaf surface temperature excess for the maximal (**b**), mean (**c**) and minimal (**d**) leaf temperatures (mean+se for each time step, i.e., every 30 min), and for the three treatments: intact leaves (grey circles), leaves covered with vegetable oil (white triangles) and leaves infested with green apple aphids (black squares). The minimal and maximal temperatures correspond to the mean temperature of the 5% coolest and hottest pixels at the leaf surface, respectively.

Temperature distributions showed a high thermal heterogeneity of leaf surfaces that encompassed a range of up to 10 °C when considering individual pixels (an illustration is given in Figure 1; see also Figure A2). In general, the temperature distribution of leaves that were covered with vegetable oil shifted toward higher surface temperatures as compared to intact leaves, and leaves with aphids were intermediate (Figure A2 and Figure A3). In all of the treatments, all the pixels of the leaf surfaces were warmer than ambient air (Figure A3). As expected, increasing air temperature caused an increase in leaf surface temperature metrics (Table 1). The aphid and vegetable oil treatments caused an increase in the maximal leaf temperature by about 2 °C at higher air temperatures when compared to intact leaves (Table 1; Figure 2a). The mean leaf temperature was on average 1.5 °C higher in leaves that were covered with vegetable oil and in leaves hosting aphids compared to intact leaves (Table 1; Figure 2b). Finally, the minimal leaf temperature was not influenced by the aphid presence or vegetable oil treatments (Table 1; Figure 2c). A similar result was obtained when taking the absolute maximal and minimal temperatures for each leaf surface instead of the mean of the 5% hottest or coldest surface temperatures (Figure A4). Although the interaction term between air temperature and the different thermal metrics was not, or was only weakly, significant (Table 1), a different dynamics was observed between the two halves of the experiment, when air temperature increased and then decreased (Figure A5). The temperature excess of the different metrics was less responsive to air temperature, and remained high during the air temperature decreasing phase (Figure A4).

### 3.2. Leaf Temperature Heterogeneity: Configuration

**Figure 3:**
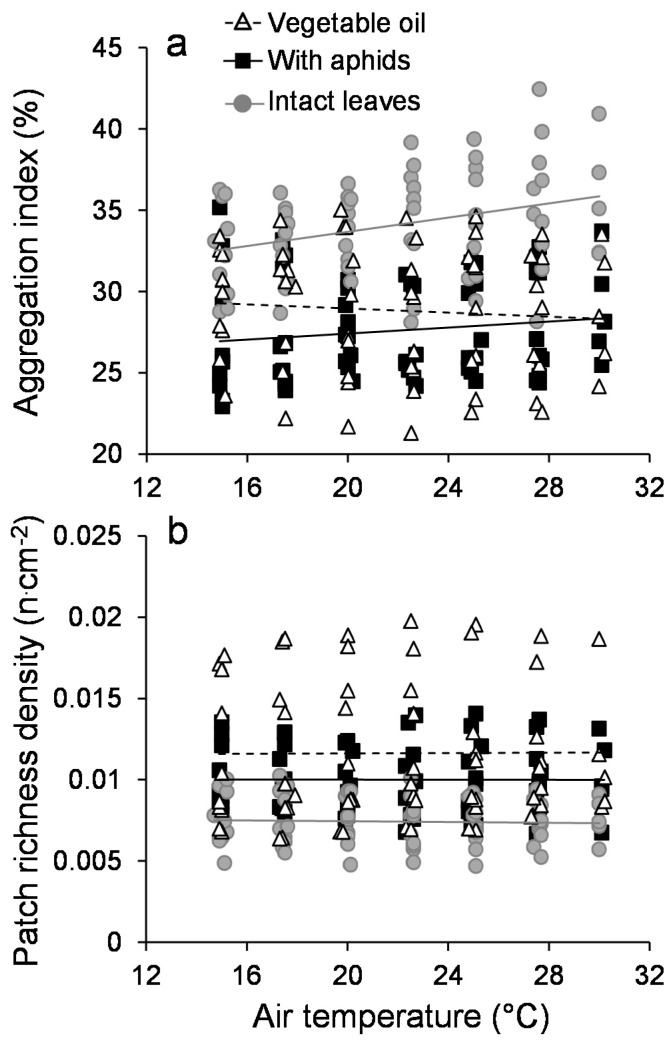
Configuration of the leaf surface thermal heterogeneity. Dynamics of (**a**) the aggregation index (AI) and (**b**) the patch richness density (PRD) as function of air temperature for intact leaves (grey circles), leaves covered with vegetable oil (white triangles) and leaves infested with green apple aphids (black squares). The AI defines the way groups of pixels with similar temperatures are arranged spatially: it is zero when similar pixels are spread across the surface, and it equals 100 when the aggregation is maximal. The PRD is the count of different temperature values (with a 0.1 °C resolution) relative to the total number of pixels in the thermographic image: it is close to zero when the diversity of temperature values is small, and it increases when every single pixel has a unique temperature value.

**Figure A6:**
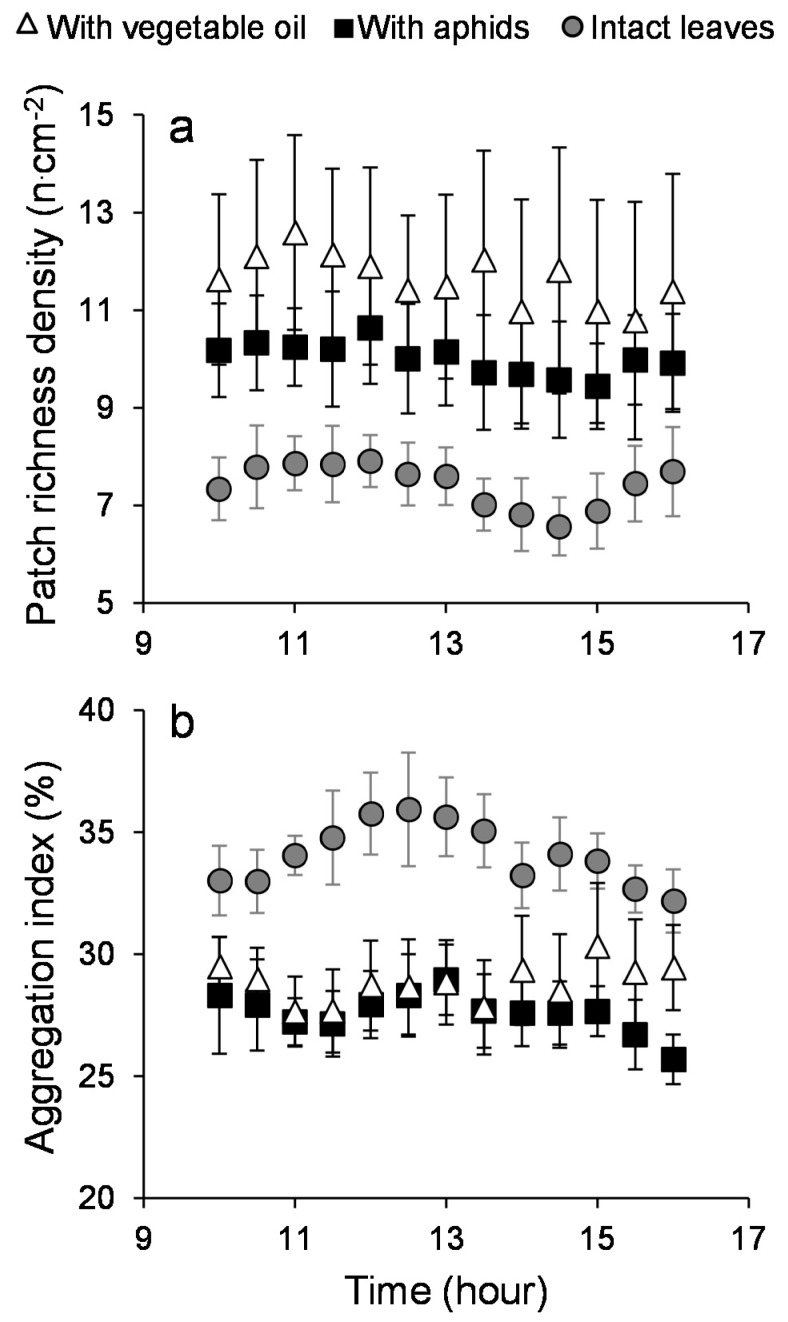
Dynamics of the patch richness density, PRD (**a**), and the aggregation index, AI (**b**), of the leaf surface temperature distributions (mean+se) in the three treatments: intact leaves (grey circles), leaves covered with vegetable oil (white triangles) and leaves infested with green apple aphids (black squares). The PRD is the count of different temperature values (with a 0.1 °C resolution) relative to the total number of pixels in the thermographic image: it is close to zero when the diversity of temperature values is small, and it increases when every single pixel has a unique temperature value. The AI defines the way groups of pixels with similar temperatures are arranged spatially: it is zero when similar pixels are spread across the surface, and it equals 100 when the aggregation is maximal.

The aggregation index (AI) and the patch richness density (PRD) were not influenced by air temperature (Table 1), and no trend was observed between the two phases of the experiment (Figure A6). The two treatments aphids and vegetable oil impacted the AI (Table 1; Figure 3a). The AI was generally lower in these two treatments when compared to intact leaves. The presence of aphids at the leaf surface did not impact the PRD significantly (Table 1; Figure 3a). Similarly, the PRD was increased in the vegetable oil and aphid treatments when compared to intact leaves (Table 1; Figure 3b). The aphid and vegetable oil treatments were quantitatively more similar to each other.

### 3.3. Aphid Position within the Leaf Thermal Pattern

**Figure 4:**
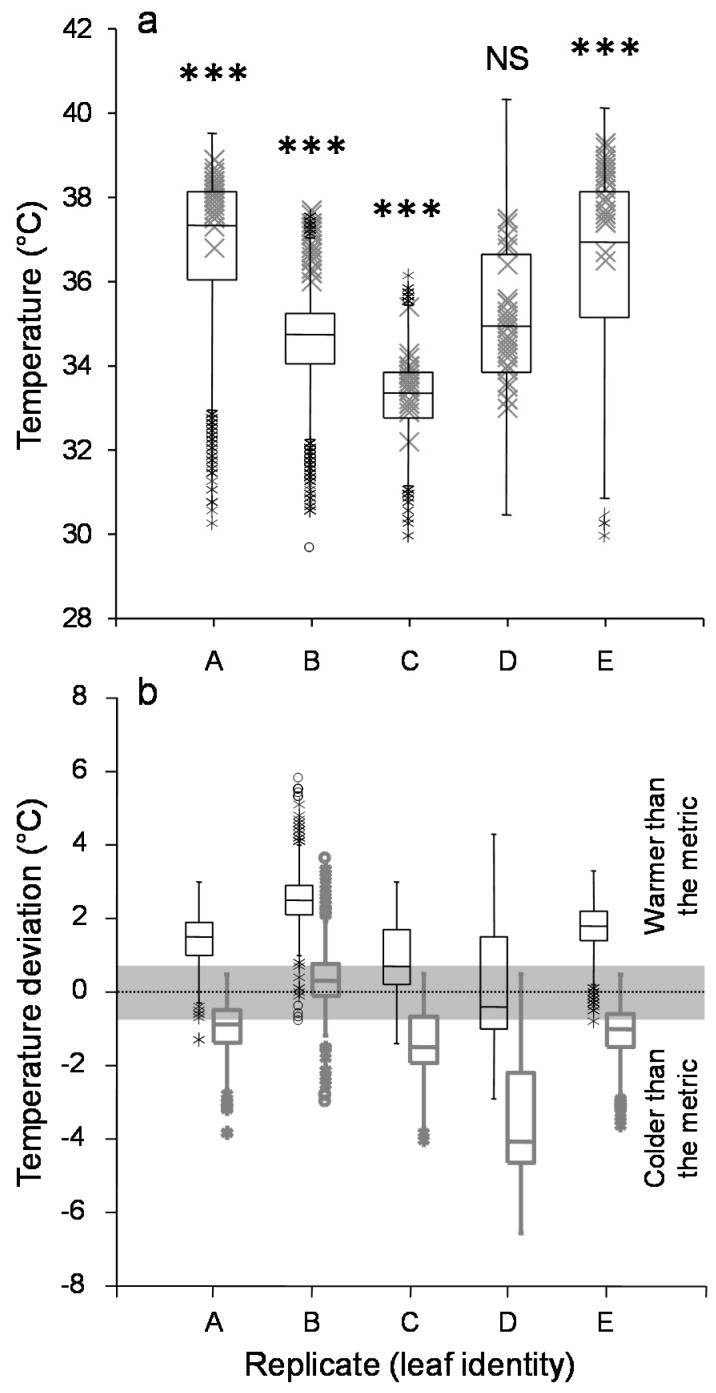
Aphid position within the leaf thermal pattern. (**a**) Overlap of the distribution of the whole leaf surface temperatures (box plot in black) and the distribution of temperatures at the position of the aphids (grey crosses) for the five replicates in the treatment with aphids on the surface of leaves. Asterisks and NS indicate a high level of significance (*p* < 0.01) and non-significance, respectively, of the difference between the two distributions for each replicate. (**b**) Deviation between the surface temperature at the position of aphids and the mean leaf surface (black box plots) or the maximal leaf surface (grey box plots) temperature distributions. The grey zone illustrates the 0.9 confidence interval beyond which a distribution is deemed to differ from a distribution centered on zero. In each box, the central mark shows the median, and the bottom and top edges (called hinges) of the box indicate the 25th and 75th percentiles, respectively. The whiskers show the range of observed values that fall within the inner fences. Values between the inner and outer fences are plotted with asterisks (outside values). Values beyond the outer fences, called far outside values, are plotted with empty circles. The fences are defined relative to the range between the two hinges (called Hpsread): Lower inner fence = lower hinge − (1.5 × (Hspread)); Upper inner fence = upper hinge + (1.5 × (Hspread)); Lower outer fence = lower hinge − (3 × (Hspread)); Upper outer fence = upper hinge + (3 × (Hspread)).

Between 30 and 34 individuals were ‘re-captured’ on the photographs at the end of the experiment. Actually, up to 50 individuals were counted by eye at the leaf surface at the end of the experiments, but some individuals were too close to each other to be discriminated from the photographs (mostly new-born individuals). Among the five replicates in the aphid treatment, the distribution of the surface temperatures at the position of the aphids differed from the overall leaf surface temperature distribution in four replicates (Figure 4a; two-sample *t*-test: *t* > 4.24 and *p* < 0.01; *t* = −0.89 and *p* = 0.38 for the fifth replicate). Indeed, aphids were systematically living on a surface warmer than the mean leaf temperature in these four replicates (Figure 4b; one-sample *t*-test against a mean temperature excess of 0 and confidence interval of 0.9: *p* < 0.01 for all; for the fifth replicate: *p* = 0.21). However, aphids still inhabited a portion of the leaf at a temperature below the maximal leaf surface temperature (mean of the 5% hottest pixels) in all of the replicates (Figure 4b; one-sample *t*-test against a maximal temperature excess of 0 and confidence interval of 0.9: *p* < 0.01 for all).

## 4. Discussion

The thermal variance of single leaf surfaces provides opportunities for behavioral thermoregulation for tiny arthropods living at the leaf surface [16,24]. Our results show that sucking insects, like aphids, slightly modify the temperature pattern of leaf surfaces during early infestation (here, after only three days of infestation). Overall, aphids induced an increase in the leaf temperature pattern mostly by shifting the temperature distribution towards higher temperature and by decreasing the aggregation of temperature patches. The maximal temperature at the leaf surface was increased by up to 2 °C, while the minimal temperature did not differ from intact leaves. The decrease in aggregation shows that the “new temperature patches” are somewhat regularly distributed across the leaf surface. In other words, the interpretation is that aphids can encounter more different temperature values when moving a few cm across a leaf surface that was attacked when compared to an intact leaf. The temperature range of a single leaf surface attacked by the aphid was around 6 °C when calculating the difference between minimal and maximal temperatures (taken as the mean of the 5% extreme values) at air temperature 30 °C, whereas it was of about 4 °C for intact leaves. These values are of the same order of magnitude as the temperature range reported for intact tree leaves, including apple [15,16]. Our results further indicate that this temperature range over individual leaf surfaces can be higher when the leaf is attacked by insect herbivores. 

The temperature range reported here when insects feed at the leaf surface could be higher under different circumstances. First, we infested leaves with a small group of aphids only three days before the experiment. This time was sufficient for the aphids to start feeding on the leaf and for the population to grow (abundances ~tripled), but a longer infestation time would exacerbate their effect on the leaf surface temperature patterns by amplifying the impact of aphids on leaf gas exchange and/or by modifying leaf shape. In addition, we did not necessarily control for the number of individuals at the leaf surface after three days (30–50 aphids), but we expect larger colonies to have a more important influence on leaf surface temperature patterns (colonies of more than 200 individuals can be observed on single leaves in apple orchards; S. Pincebourde, personal observation). Secondly, while we manipulated air temperature during the experiment, the other environmental factors were fixed, in particular, irradiance (250 W·m^−2^) and wind speed (0.4 m·s^−1^) within the climatic chamber. Irradiance and wind speed are known to influence mean leaf temperature [18], as well as the leaf surface heterogeneity [16]. Increasing irradiance generates higher temperature ranges at the leaf surface [16], and our conditions were moderate when compared to the maximum irradiance that can be reached in the field (>1000 W·m^−2^). Saudreau et al. [16] reported temperature ranges of up to 20 °C for intact leaves under high irradiance (800 W·m^−2^) and low wind. By contrast, increasing wind speed homogenizes the surface temperature of leaves and brings the mean leaf temperature close to ambient air temperature [16]. Therefore, the complex interactions between all of the environmental factors should be considered before applying our results to other conditions.

The influence of aphids on leaf surface temperature may be related to their feeding activity (phloem feeders). The population increased during the three-day infestation period, indicating that aphids were feeding on the leaf surface. We cannot rule out, however, the possible influence of the plant response to the sole insect presence at the surface, as shown already for other species, in particular, during egg deposition [43] or when the insect touches trichomes [44]. We cannot exclude either that the presence of aphids might locally modify the leaf boundary layer resistance with consequences for the energy budget of at least portions of the leaf surface, although such an effect would be expected to be small in our experiments given the size of an aphid relative to the size of the boundary layer of non-infected leaves (see [45] for insect eggs). Also, we probably had too few individuals that were grouped together to potentially limit water vapor diffusion across the leaf boundary layer. Partitioning these two effects (insect feeding and presence) requires a treatment with aphids that do not feed on leaf tissues, which is practically impossible. Deposition of honeydew on the leaf surface may also block the stomata, thus reducing evapotranspiration and increasing leaf temperature, although we did not observe honeydew on the surface of leaves after three days of infestation. Finally, the shift in leaf temperature pattern under aphid attack is not related to the leaf initiating death processes. We never observed apple leaves to dry and to die even after two months of aphid infestation whether in the field or under greenhouse conditions.

The mechanisms at play when aphids induce a shift in the leaf surface temperature distribution may be linked to the stomatal behavior of the leaf, whether the plant responds to feeding activity or to insect presence alone. Our results show that the temperature pattern of aphid-infested leaves is similar to the pattern of leaves with the underside covered with vegetable oil to inhibit transpiration. Therefore, green apple aphids probably induce stomatal closure and a decrease in transpiration rate when feeding on leaf tissues during early infestation. A decrease in leaf transpiration rate was observed during feeding with the aphid *Acyrthosiphon pisum* [34]. By contrast, other aphid species (e.g., *Aphis gossypii* and *A. fabae*) induce an increase in the leaf transpiration rate [35,36], and therefore, they should cause a decrease in mean leaf temperature. The explanation of these inter-specific differences is not clear. During feeding, the aphid usually inserts its stylet through the leaf epidermis and probes across leaf tissues until it finds the phloem [46]. The amount of laceration that is inflicted by aphid stylets on plant tissue is generally negligible and internal damage is more linked to chemical interactions rather than to physical injuries [47]. For example, aphids can release immuno-suppressive proteins via their saliva during feeding [48], but their influence on stomatal behavior has never been investigated. A high diversity of effector proteins is expected among aphid species, which may help explain the discrepancies in effect on transpiration rates across species. We speculate that the direction of the effect of aphids on the leaf surface temperature patterns could be correlated to the nature of the effector proteins that are delivered by the insect within plant tissues. However, currently no data are available to test this hypothesis.

The consequences of the aphid-induced shift in thermal pattern for the plant eco-physiology are expected to be significant, even after only 3 days of infestation. The increase in maximal temperature at the leaf surface, bringing patches above 40 °C in our experiment, may superimpose thermal stress and herbivore-induced stress locally on leaf tissues, depending on the environmental conditions. Biochemical signals of thermal stress can be measured as soon as leaf temperature reaches 40 °C [49]. In addition, the photosynthesis rate is likely to be negatively affected during early infestation by *A. pomi*, similarly to transpiration rate. At the scale of a single leaf, local changes in photosynthesis were observed when an insect chews on leaf tissues [29,32] or when an insect induces a gall on a leaf (see [50]). However, we are not aware of a similar study on the local impact (e.g., cm scale) of phloem feeders on photosynthesis. In general, the photosynthetic rate of intact leaves declines beyond surface temperature of 30 °C in temperate species [51]. Therefore, the 2 °C increase in the hottest parts of the leaf should lower photosynthesis locally. The performance of a leaf is expected to be altered by the change in thermal pattern, but the plant may also compensate (partially) for the loss in carbon assimilation via increased water use efficiency, as shown for a leafminer species in apple [27].

*Aphis pomi* aphids were positioned in leaf portions where the temperature varies between the maximal and the mean leaf surface temperature—we stress again that lower and upper leaf surfaces show similar temperature patterns in apple [24]. The warmest portions of a leaf are found far from leaf edges, toward the center of a leaf surface, and around main and secondary veins (see Figure 1), where most aphids are positioned. Therefore, because the aphids are more often located at temperatures above mean and below maximum (sometimes close to maximum), one can infer that the aphid-induced increase in maximal surface temperature could be felt by aphids. Our experimental conditions were not stressful a priori for both the plant and the aphids, with a moderate irradiance level and fluctuating air temperature up to 30 °C for a short period of daytime. Under these environmental conditions at midday, aphids were found systematically on tissues at temperature above 32 °C and up to 39 °C. Constant temperature experiments showed that the optimal temperature for development in *A. pomi* is around 30 °C and that development stops at temperatures above 35 °C [52]. Although constant temperature experiments do not reflect the thermal requirements under fluctuating environments [53], it is possible that aphids suffered from thermal stress at midday during our experiments. The 2 °C increase in maximal surface temperature is significant relative to this temperature range inducing thermal stress (30–35 °C), thereby increasing the intensity of thermal stress for the aphid. Behavioral thermoregulation is not known in this species, but other species showed great abilities to move within the plant, from leaf to leaf, to avoid exposure to high temperatures [54,55]. Behavioral thermoregulation within the single leaf surface, however, is not known in any aphid species. Like many aphid species, *A. pomi* establishes near or on the main and secondary veins of the leaf to feed on the phloem. Feeding need probably constrains the ability to thermoregulate behaviorally, but this remains challenging to investigate [56]. Moreover, the nutritional quality of plant tissues in the hottest spots of the leaf may be decreased, as shown for long term impact of temperature in woody species [57], but here again, more research is needed to determine the extent to which leaf surface thermal heterogeneity translates into heterogeneity in leaf nutritional quality.

Aphids potentially increase the opportunities for behavioral thermoregulation, since a wider temperature range is available (although surface temperature is less patchy), at least within the limits of the conditions tested here (e.g., no water stress and low water vapor deficit, moderate air temperature and irradiance). However, this effect may not increase their resilience to high temperatures for at least two reasons. First, we found that aphids increase maximal leaf surface temperature, but not the minimal temperature. Aphids create new patches of elevated surface temperature, but not cooler patches. Therefore, aphids cannot escape the heat more than on intact leaves. Secondly, we manipulated air temperature within a range of moderate values (15–30 °C) but higher air temperatures (38 °C) were shown to significantly homogenize the surface temperature of single leaves [24]. The insect-induced modification of the leaf thermal patterns may allow for aphids to find temperature close to their physiological optimum under moderate conditions, although the extent to which aphids can move across leaf surfaces in response to temperature remains unknown for most aphids, and unlikely in *A. pomi* from our experiments. It is not known either if, under high air temperature, aphids influence the minimal surface temperature to the point that they cause colder-than-ambient patches, allowing for them to escape the heat. These inter-relationships between microhabitat heterogeneity, movements of organisms, and environmental changes constitute the basal mechanism in the response of ectotherms to environmental changes [58,59]. In this system, the feedback effect of feeding activity by insects on their host plant can be important and should be quantified to better predict the response of phytophagous insects to environmental changes.

## Figures and Tables

**Table 1:** Analysis of co-variance (ANCOVA, type II tests) of the response of maximal (T_max_), mean (T_mean_) and minimal (T_min_) leaf surface temperatures, as well as aggregation index (AI) and patch richness density (PRD) to the different treatments (presence of aphids, vegetable oil or intact leaves) with air temperature as a covariable. The replicate (leaf identity) was set as a random factor. *p*-Values below the threshold of 0.05 are indicated in bold. Legend: T_air_, air temperature; Sum sq, sum of squares; df, freedom degrees.

Variable	Effect	Sum sq	df	*F*-value	*p*-Value
T_max_	Treatment	80.9	2	22.83	**<0.01**
	T_air_	4548.6	1	2565.42	**<0.01**
	Treatment*T_air_	9.9	2	2.79	0.064
	Residuals	333.3	188		
T_mean_	Treatment	35.4	2	13.91	**<0.01**
	T_air_	4755	1	3732.65	**<0.01**
	Treatment*T_air_	7.5	2	2.93	0.056
	Residuals	239.5	188		
T_min_	Treatment	4.8	2	2.66	0.073
	T_air_	4840.9	1	5371.81	**<0.01**
	Treatment*T_air_	5.7	2	3.14	**0.046**
	Residuals	169.4	188		
AI	Treatment	1542.4	2	67.32	**<0.01**
	T_air_	30.7	1	2.68	0.103
	Treatment*T_air_	57.4	2	2.50	0.084
	Residuals	2153.7	188		
PRD	Treatment	5.8 × 10^−4^	2	34.74	**<0.01**
	T_air_	3.0 × 10^−7^	1	3.1 × 10^−3^	0.955
	Treatment*T_air_	2.5 × 10^−6^	2	0.01	0.985
	Residuals	1.6 × 10^−3^	188
